# Characterization of Intestinal and Hepatic CYP3A-Mediated Metabolism of Midazolam in Children Using a Physiological Population Pharmacokinetic Modelling Approach

**DOI:** 10.1007/s11095-018-2458-6

**Published:** 2018-07-30

**Authors:** Janneke M. Brussee, Huixin Yu, Elke H. J. Krekels, Semra Palić, Margreke J. E. Brill, Jeffrey S. Barrett, Amin Rostami-Hodjegan, Saskia N. de Wildt, Catherijne A. J. Knibbe

**Affiliations:** 1 Division of Systems Biomedicine and Pharmacology, Leiden Academic Centre for Drug Research (LACDR), Leiden University, Leiden, the Netherlands; 2 Present Address: Novartis, Basel, Switzerland; 3 Present Address: Netherlands Cancer Institute (NKI), Amsterdam, the Netherlands; 4 Department of Pharmaceutical Biosciences, Uppsala University, Uppsala, Sweden; 5 Translational Informatics, Sanofi, Bridgewater, New Jersey USA; 6 Department of Pediatrics, Division of Clinical Pharmacology & Therapeutics, Children’s Hospital of Philadelphia, Philadelphia, Pennsylvania USA; 7 Centre for Applied Pharmacokinetic Research, University of Manchester, Manchester, UK; 8 Simcyp Limited (A Certara Company), Sheffield, UK; 9 Intensive Care and Department of Pediatric Surgery, Erasmus MC - Sophia Children’s Hospital, Rotterdam, the Netherlands; 10 Department of Pharmacology and Toxicology, Radboud University Medical Centre, Nijmegen, the Netherlands; 11 Department of Clinical Pharmacy, St. Antonius Hospital, Nieuwegein, the Netherlands

**Keywords:** CYP3A, extraction ratio, absorption, first-pass metabolism, gut wall, liver, pediatrics

## Abstract

**Purpose:**

Changes in drug absorption and first-pass metabolism have been reported throughout the pediatric age range. Our aim is to characterize both intestinal and hepatic CYP3A-mediated metabolism of midazolam in children in order to predict first-pass and systemic metabolism of CYP3A substrates.

**Methods:**

Pharmacokinetic (PK) data of midazolam and 1-OH-midazolam from 264 post-operative children 1–18 years of age after oral administration were analyzed using a physiological population PK modelling approach. In the model, consisting of physiological compartments representing the gastro-intestinal tract and liver,intrinsic intestinal and hepatic clearances were estimated to derive values for bioavailability and plasma clearance.

**Results:**

The whole-organ intrinsic clearance in the gut wall and liver were found to increase with body weight, with a 105 (95% confidence interval (CI): 5–405) times lower intrinsic gut wall clearance than the intrinsic hepatic clearance (i.e. 5.08 L/h (relative standard error (RSE) 10%) *versus* 527 L/h (RSE 7%) for a 16 kg individual, respectively). When expressed per gram of organ, intrinsic clearance increases with increasing body weight in the gut wall, but decreases in the liver, indicating that CYP3A-mediated intrinsic clearance and local bioavailability in the gut wall and liver do not change with age in parallel. The resulting total bioavailability was found to be age-independent with a median of 20.8% in children (95%CI: 3.8–50.0%).

**Conclusion:**

In conclusion, the intrinsic CYP3A-mediated gut wall clearance is substantially lower than the intrinsic hepatic CYP3A-mediated clearance in children from 1 to 18 years of age, and contributes less to the overall first-pass metabolism compared to adults.

**Electronic supplementary material:**

The online version of this article (10.1007/s11095-018-2458-6) contains, which is available to authorized users.

## Introduction

Differences in drug absorption and first-pass metabolism have been reported in children compared to adults (1–3). These differences may for instance result from a smaller intestinal surface area in children (2) and altered permeability across age (1). In addition, besides gastric emptying time, intestinal transit time, the production of bile fluid (3) and organ blood flow to intestines and liver that may be altered in children (4), intestinal and hepatic drug metabolizing enzyme activity may be different from those in adults. For first-pass metabolism, the activity of both intestinal and hepatic enzymes is of relevance, while for systemic clearance the activity of hepatic enzymes is important with activity of intestinal enzymes probably being of negligible influence.

With cytochrome P450 (CYP) being an enzyme family involved in metabolism of many drugs (5), this study focuses on CYP3A enzymes, as they are abundant in both intestine and liver (6,7). From the available *in vitro* and *in vivo* studies on the ontogeny of CYP3A in children (8–10), there is some evidence that the maturation and regulation of these enzymes in the gut wall and liver may differ (11). However, the translation of enzyme activity in gut wall and liver to intestinal and hepatic clearance is complex, because other parameters like organ blood flow, organ size and other physiological parameters need to be taken into account (4,12). In order to distinguish between intestinal and hepatic clearance (and their maturation), a combination of mechanistic and empirical models can be useful, as was shown in adults before (13,14). This hybrid of approaches seems necessary as for full physiologically-based PK models in children not all parameters are always available and/or identifiable, while empirical models may lack direct physiological interpretation. The latter is particularly problematic, because both hepatic and intestinal metabolism contribute to first-pass metabolism. As such, the combination of PBPK concepts with population modelling using PK data from children enables incorporation of prior knowledge of the system, while obtaining more insight into the system by parameter estimation based on reverse translation of the observed clinical data (15).

In this study, the aim is to characterize both intestinal and hepatic CYP3A-mediated metabolism of midazolam in children between 1 and 18 years of age. We will adopt the above described physiological population PK modelling approach, in which we account for known changes in the physiology of the gastrointestinal tract, and use the available PK data from the population, to estimate the whole-organ intrinsic intestinal and hepatic CYP3A-mediated midazolam clearance in children. For this analysis, we had access to data from a clinical study in which the CYP3A substrate midazolam which is considered a probe drug for CYP3A (16), was administered to children pre-operatively, and in which both midazolam and the CYP3A-mediated metabolite (i.e. 1-OH-midazolam) concentrations were available.

## Methods

### Data

In 873 plasma samples from 266 patients of the Children’s Hospital of Philadelphia, PA, midazolam and 1-OH-midazolam concentrations were measured (17). The children received a median dose of 10 mg (range 3–15 mg) of midazolam as oral suspension pre-operatively. Subjects include children (150 boys/116 girls) between 1 and 18 years of age (median 7 years), with a median body weight of 27.2 kg (range 9.1–137.6 kg), who fit the criteria I or II of the American Society of Anesthesiologist’s (ASA) classification, undergoing surgery. In 31 patients, midazolam and its primary metabolite were densely sampled around 0.25, 0.5, 1, 1.5, 2, 3, 4, 6, 8, 10 and 22 h after dose administration with a median of 10 samples per patient (range 8–11), and in 235 patients, samples were sparsely collected for analysis at different time points post-dose (median 2, range 1–3 samples/patient) with most samples within the first four hours after dose administration (figure S1). Data from two 14-year old patients in the sparsely sampled group were excluded as their body weight was <12 kg. The patient groups who were in the dense and sparse sampled study were comparable in age and weight distribution (Table SI). Patient height and body surface area, required for some of the covariate relationships in the model, were derived from recorded age and body weight information (equations in supplemental material).

Blood concentrations (*B*) were estimated based on the measured plasma concentrations (*P*) using eq. 1 (18):

1 $$ B:P=1+\left[ Hem\times \left({f}_u\times {K}_p-1\right)\right] $$

In which Hem is the hematocrit based on population values reported in literature (19) ranging from 0.36 in 1- and 2-year-old infants up to 0.41–0.43 in adolescents of 12–18 years of age (Table I), the f_u_ is the fraction unbound in plasma and K_p_ is the unbound partition coefficient of the red blood cells to plasma (assumed to be constant between adults and children) (18). The fraction unbound in plasma for both midazolam and 1-OH-midazolam were calculated based on the formula of McNamara and Alcorn (20):

2 $$ {f}_{\mathrm{u}, pediatric}=\frac{1}{1+\frac{\left(1-{f}_{u, adult}\right)\times {\left[P\right]}_{pediatric}}{{\left[P\right]}_{adult}\times {f}_{u, adult}}} $$

where f_u,pediatric_ and f_u,adult_ are the fraction unbound of the drug in plasma for children and adults respectively and [P]_pediatric_ and [P]_adult_ are the plasma albumin concentrations in children and adults respectively, assuming exclusive binding to albumin for midazolam and its metabolite. The fractions unbound of midazolam and 1-OH-midazolam in plasma in adults were reported in literature (21,22). The albumin concentrations [P]_pediatric_ are calculated based on the formula of Johnson *et al*. (12):

3 $$ {\left[\mathrm{P}\right]}_{\mathrm{pediatric}}\left[\mathrm{g}/\mathrm{L}\right]=1.1287\times \ln \left(\mathrm{Age}\left[\mathrm{yr}\right]\right)+33.746 $$

**Table I:** Parameter Values for System Specific and Drug Specific Parameters Included in the Physiological Population PK Model

Parameter name (unit)	Parameter symbol	Formula for calculation	Value	References
Tissue volumes (L)				
Liver	V_h_	0.722 × BSA^1.176^	–	(25)
Portal vein	V_pv_	–	0.0052	(26)
Small intestine	V_in_	0.0467 × age + 0.0901	–	(4)
Tissue blood flows (L/h)				
Cardiac output	CO	BSA × (110 + 184.974 × (e^-0.0378 × age^ - e^-0.24477 × age^))	–	(27)
Hepatic blood flow	Q_h_	0.28 × CO (♀) 0.255 × CO (♂)	–	(27)
Portal vein Hepatic artery	Q_pv_ Q_ha_	0.75 × Q_h_ 0.25 × Q_h_	–	(12,52)
Small intestine Mucosa Microvilli	Q_in_ Q_muc_ Q_villi_	0.4 × Q_h_ 0.8 × Q_in_ 0.6 × Q_muc_	–	(30,49)
Plasma proteins				
Plasma albumin concentration (g/L)	P_pediatric_ P_adult_	1.1287 × ln(age) + 33.746	- 37.7	(12)
Hematocrit (%)	Hem_1-2y_ Hem_3-6y_ Hem_7-12y_ Hem_12-18y,♀_ Hem_12-18y,♂_	–	0.36 0.37 0.40 0.41 0.43	(19)
Midazolam				
Fraction absorbed	F_a_	–	1	(29)
Absorption rate constant (h^−1^)	K_a_	–	4.16	–
Blood: plasma ratio	B:P ratio	1 = [*Hem* × (*f*_*u*_ × *K*_*p*_ − 1)]with *K*_*p*_ = 1	–	(18)
Fraction unbound in gut	F_u,G_	–	1	–
Fraction unbound in plasma	F_u,plasma_ F_u,adult_	$\frac{1}{1+\frac{\left(1-{f}_{u, adult}\right)\times {\left[P\right]}_{pediatric}}{{\left[P\right]}_{adult}\times {f}_{u, adult}}}$	0.0303	(20) (21)
Permeability through the enterocyte (L/h)	CL_perm_	CL_perm_ = P_eff,man_ × A with P_eff,man_ = 4.4 × 10^−4^ cm/s	–	(30)
Intestinal surface area (dm^2^)	A	2πr(r + h) with radius r = ½ × (0.016 × BSA + 0.0159) and length h = 2.56 × BSA + 2.95	–	(12)
1-OH-midazolam				
Blood: plasma ratio	B:P ratio	*B* : *P* = 1 + [*Hem* × (*f*_*u*, *M*_ × *K*_*p*_ − 1)] with *K*_*p*_ = 1	–	(18)
Fraction unbound in plasma	F_u,M,pediatric_ F_u,M,adult_	$\frac{1}{1+\frac{\left(1-{f}_{u,M, adult}\right)\times {\left[P\right]}_{pediatric}}{{\left[P\right]}_{adult}\times {f}_{u,M, adult}}}$	0.106	(20) (22)

Age is expressed in years. A is the intestinal surface area in dm^2^. BSA is the body surface area in m^2^. P_eff,man_ is the effective intestinal permeability per unit surface area (dm/h). WT is body weight in kg

♀ female, ♂ male

Measurements below the lower limit of quantification were discarded according to the M6 method (23) (*n* = 4 (0.5%) and *n* = 5 (0.6%) of midazolam and 1-OH-midazolam measurements respectively).

## Model Development

### Structural Model

**Fig. 1:**
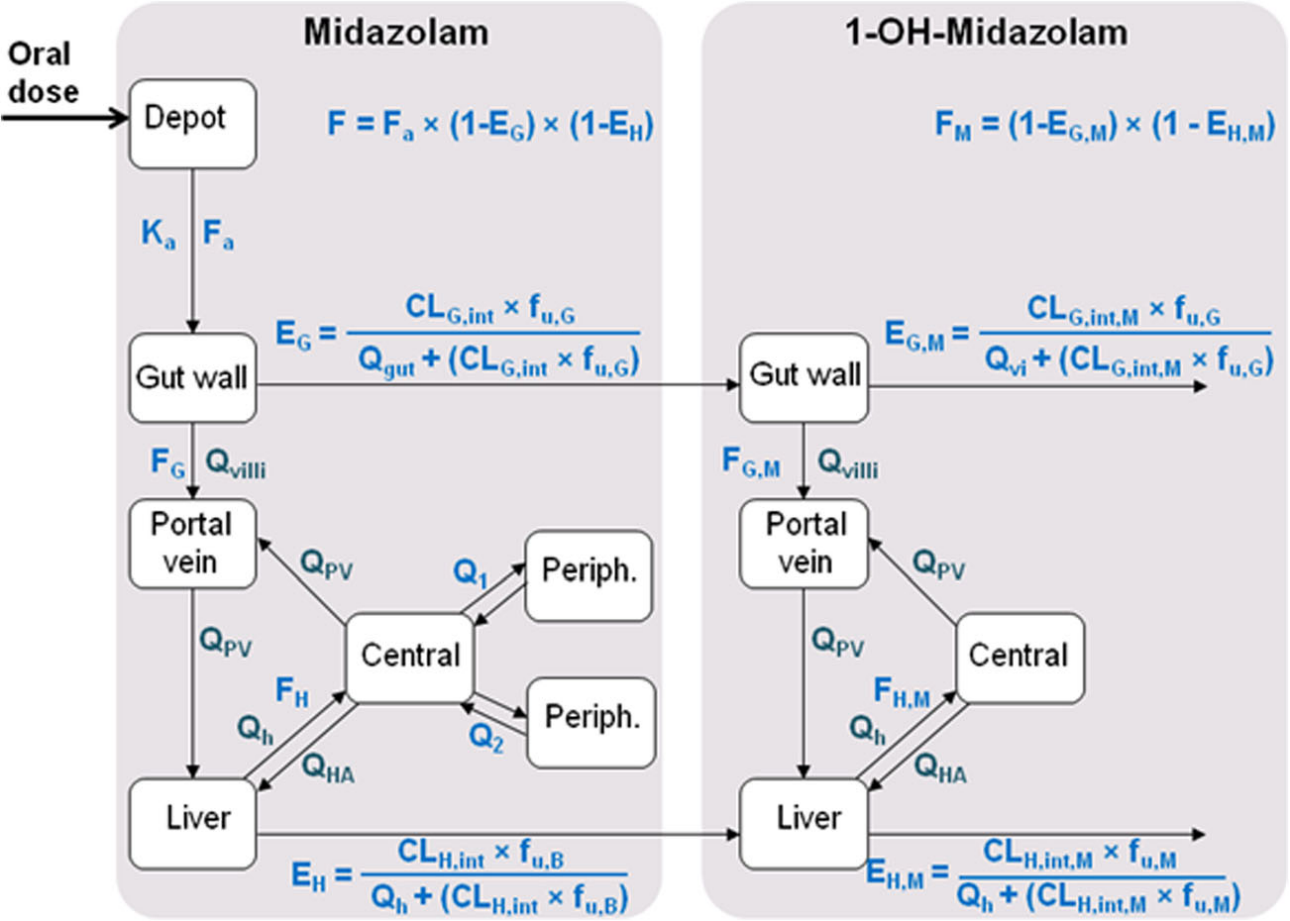
Schematic representation of the physiological population PK model for midazolam and the metabolite 1-OH-midazolam. The extraction of midazolam is defined by the well-stirred model in the liver and the ‘Q_gut_’ model in the gut wall. E = extraction ratio, F = bioavailability in the gut wall (gut, G) and the liver (hepatic, H). CL_int_ is the whole-organ intrinsic clearance in the gut wall and liver, Ka indicates the absorption rate constant and the fraction unbound in blood and gut wall are described with f_u,B_ and f_u,G_ respectively. Blood flows are represented by Q; in the micro villi (Q_villi_), portal vein (Q_PV_), hepatic artery (Q_HA_) and liver (Q_h_). Distribution between central and peripheral (Periph.) compartments is estimated by inter-compartmental clearance Q_1_ and Q_2_ for the two peripheral compartments for midazolam. Parameters describing the metabolite are indicated with the subscript M.

The physiological population PK analysis was performed using NONMEM version 7.3 (ICON, Globomax LLC, Ellicott, MD, USA) based on first-order conditional estimation with interaction, and for visualization of data Pirana 2.9.0, R (version 3.3.1), and R-studio (version 0.98.1078) were used. A physiological population pharmacokinetic (PK) model, earlier developed and applied by Yang *et al*. (24), Frechen *et al*. (13) and Brill *et al*. (14) to describe midazolam PK data in adults, was now applied to describe the midazolam PK data in children 1–18 years of age (Fig. 1). This model includes physiological compartments representing the gut wall, the portal vein and the liver, and also empirical central and peripheral compartments for midazolam and 1-OH-midazolam distribution, representing the blood circulation and fast equilibrating tissue, and peripheral slow equilibrating tissues (13,24). Based on literature, central and peripheral volumes were linearly scaled based on body weight from a 76 kg healthy adult with volumes of 20.4 L/76 kg, 55.2 L/76 kg and 79.1 L/76 kg for the central and two peripheral volumes for midazolam respectively and with a volume of 65.7 L/76 kg for 1-OH-midazolam (13). The fraction midazolam metabolized into 1-OH-midazolam was assumed 100% (Table II).

**Table II:** Parameter Estimates of the Final Physiological Population PK Model

Parameter definition	Parameter (unit)	Value (RSE%) [shrinkage %]	Bootstrap median	Bootstrap 90% CI	70-kg individual
Midazolam					
Intrinsic hepatic clearance CL_H,int,i_ = CL_H,int,16kg_ × (WT/16)^k1^	CL_H,int,16kg_ (L/h)	527.0 (7%)	601.4	523.5–748.6	1057
Exponent	k1	0.472 (16%)	0.425	0.206–0.554	0.472
Intrinsic gut wall clearance CL_G,int,i_ = CL_G,int,16kg_ × (WT/16)^k2^	CL_G,int,16kg_ (L/h)	5.08 (10%)	4.93	3.32–6.17	16.7
Exponent	k2	0.807 (10%)	0.881	0.622–1.27	0.807
Volume of distribution (central) V_c,i_ = V_c,76kg_ × (WT/76)^k3^	V_c,76kg_ (L)	20.4 fix	–	–	18.8
Volume of distribution (two peripheral compartments) V_p,i_ = V_p,76kg_ × (WT/76)^k3^	V_p1,76kg_ (L) V _p2,76kg_ (L)	55.2 fix 79.1 fix	- -	–	50.8 72.9
Exponent	k3	1 fix	–	–	1
Inter compartmental clearance Q_cp1,i_ = V_cp1,16kg_ × (WT/16)^k4^	Q_cp1_ (L/h)	14.9 (19%)	14.9	8.9–25.9	57.9
Exponent	k4	0.92 (21%)	1.03	0.796–1.65	0.92
Inter compartmental clearance (2nd peripheral compartment)	Q_cp2_ (L/h)	7.5 (10%)	7.7	6.3–11.2	7.5
1-OH-midazolam (M)					
Fraction midazolam metabolized into 1-OH-midazolam	f_M_	1 fix	–	–	1
Intrinsic hepatic clearance CL_H,int,M,i_ = CL_H,int,M,16kg_ × (WT/16)^k5^	CL_H,int,M,16kg_ (L/h)	235.0 (6%)	236.2	219.6–269.6	614.2
Exponent	k5	0.651 (9%)	0.615	0.451–0.750	0.651
Intrinsic gut wall clearance CL_G,int,M,i_ = k6 × CL_G,int,i_	k6	18.4 (12%)	19.2	10.6–198.2	18.4
Volume of distribution V_M,i_ = V_M,76kg_ × (WT/76)^k7^	V_M,76kg_ (L)	65.7 fix	–	–	60.5
Exponent	k7	1 fix	–	–	1
Inter individual variability (variance)					
Intrinsic hepatic clearance	ω^2^ CL_H,int_	0.25 (13%)[25%]	0.24	0.16–0.32	–
Intrinsic gut wall clearance	ω^2^ CL_G,int_	1.20 (13%)[13%]	1.25	1.04–1.56	–
Inter compartmental clearance	ω^2^ Q_cp1_	1.05 (35%)[42%]	1.05	0.26–1.85	–
	ω^2^ Q_cp2_	1.06 (31%)[46%]	1.06	0.68–1.79	–
Intrinsic hepatic clearance 1-OH-midazolam (M)	ω^2^ CL_H,int,M_	0.13 (18%)[31%]	0.13	0.08–0.19	–
Residual variability (variance)					
Proportional error (Midazolam)		0.166 (8%)[19%]	0.169	0.150–0.199	–
Additive error (Midazolam), nmol/L		0.001 fix	–	–	–
Proportional error (1-OH-midazolam)		0.292 (11%)[9%]	0.271	0.225–0.309	–
Additive error (1-OH-midazolam), nmol/L		0.528 (10%)[9%]	0.454	0.130–0.826	–

RSE: relative standard error. CI: the 5th–95th percentiles are shown, indicating a 90% confidence interval. Bootstrap *n* = 250. Inter-individual and residual variability values are shown as variance estimates. Intrinsic clearance values are reported for the whole organ

In the physiological compartments, tissue volumes and blood flows in children were based on literature data. The hepatic volume (V_h_) was calculated based on body surface area (BSA, m^2^) (25):

4 $$ {\mathrm{V}}_{\mathrm{h}}\left[\mathrm{L}\right]=0.722\times {\mathrm{BSA}}^{1.176} $$

Volume of the portal vein was assumed to be equal to the reported value of 5.2 mL in adults (26). To calculate the volume of the intestines, a regression line was derived from data published by Björkman (4):

5 $$ {\mathrm{V}}_{\mathrm{in}}\left[\mathrm{L}\right]=0.0467\times \mathrm{Age}\left[\mathrm{y}\right]+0.0901 $$

To calculate organ weight, the organ volumes are multiplied by the organ density of 1040 g/L (4). To compare with adult values, organ weight in adults is calculated assuming organ volumes of 1 L (13) and an organ density of 1040 g/L.

**Fig. 2:**
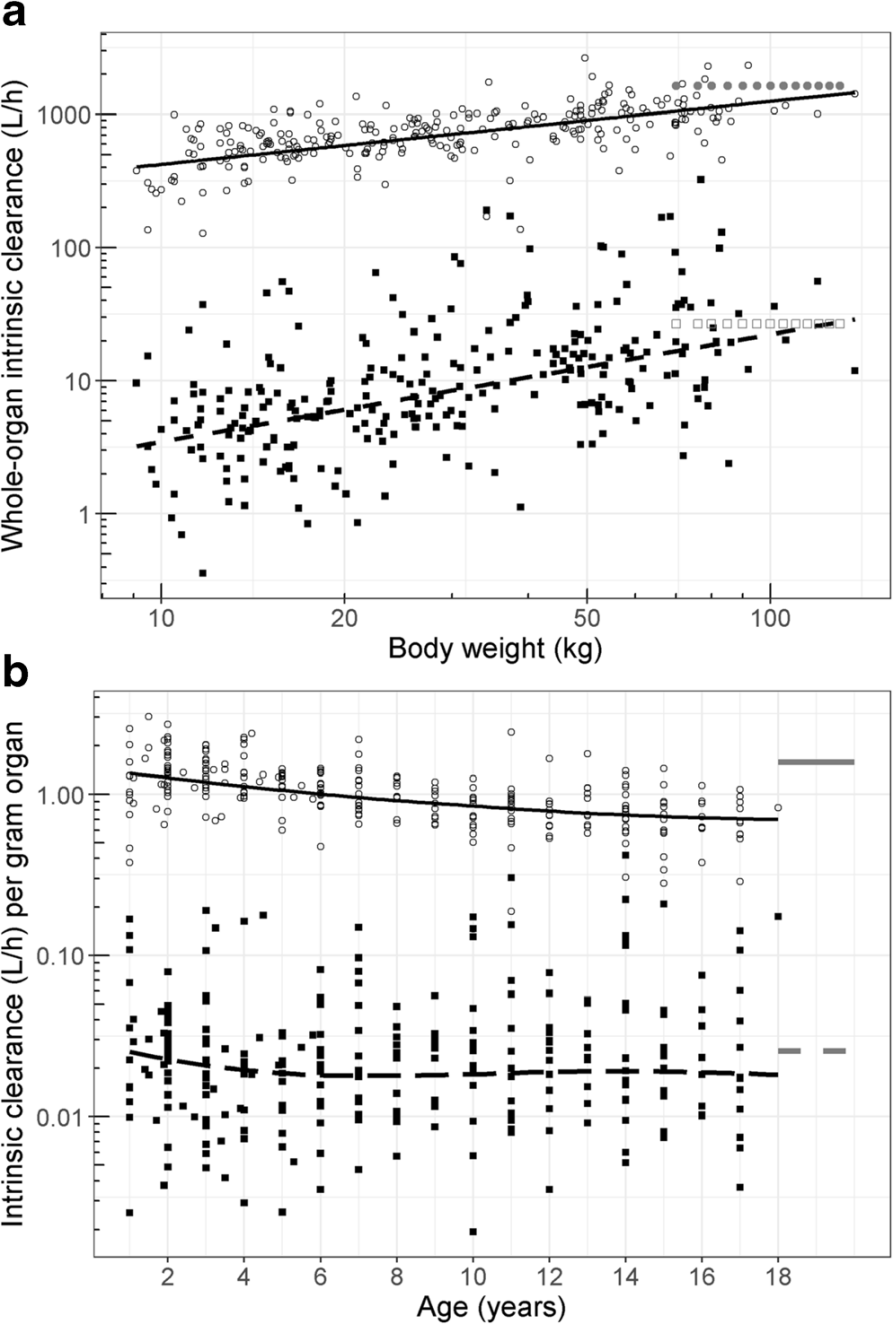
First-pass metabolism parameters in children. (**a**) Intrinsic whole-organ intestinal (■) and hepatic (○) clearance *versus* body weight, both individually predicted (symbols) and the population predictions (lines) for children in our study. Also illustrated are the reported literature values of 26.7 and 1640 L/h for intestin al () and hepatic () clearance in adults, respectively (13). (**b**) Intrinsic gut wall (■) and hepatic (○) clearance per gram of organ *versus* age for children in our study and for adults(), both individually predicted (symbols) and a loess curve of the population predictions (lines) for children in our study.

The hepatic blood flow (Q_h_) was assumed to be a fixed percentage of the cardiac output (CO), which was calculated based on BSA (27):

6 $$ \mathrm{CO}=\mathrm{BSA}\times \left(110+184.974\times \left({\mathrm{e}}^{-0.0378\times age}-\mathrm{e}-0.24477\times age\right)\right) $$

Other tissue blood flows were assumed to be proportional to the hepatic blood flow (Table I). The relationship between plasma protein binding, intestinal surface area, tissue volumes, and organ blood flows and body weight for the individual patients in this study are depicted in figure S2. For all physiological parameters in Table I, population values were used without inter-individual variability or uncertainty.

The absorption rate constant (k_a_) for midazolam could not be estimated, and was therefore fixed at 4.16 h^−1^, yielding peak concentrations to be reached round 30 min (t_max_), which is in agreement with the observed t_max_ and other reported literature values (28). The oral bioavailability (F_total_) was calculated using:

7 $$ {\mathrm{F}}_{\mathrm{total}}={\mathrm{F}}_{\mathrm{a}}\times {\mathrm{F}}_{\mathrm{g}}\times {\mathrm{F}}_{\mathrm{h}} $$

in which F_a_ is the fraction absorbed, which is assumed 1 for midazolam (29), F_g_ is the gut wall bioavailability, equal to 1 minus the gut wall extraction ratio (E_g_), and F_h_ is the hepatic bioavailability, which is equal to 1 minus the hepatic extraction ratio (E_h_). The hepatic extraction ratio of midazolam (E_H_) and 1-OH-midazolam (E_H,M_) were defined by the well-stirred model:

8 $$ {E}_H=\frac{CL_{H,\mathit{\operatorname{int}}}\times {f}_{u,B}}{Q_h+\left({CL}_{H,\mathit{\operatorname{int}}}\times {f}_{u,B}\right)} $$

where CL_H,int_ is the estimated intrinsic hepatic clearance (whole-organ), f_u,B_ is the fraction unbound in the blood and Q_h_ is the hepatic blood flow. The gut wall extraction ratio is described using the Qgut model (30):

9 $$ {E}_G=\frac{CL_{G,\mathit{\operatorname{int}}}\times {f}_{u,G}}{{\mathrm{Q}}_{gut}+\left({CL}_{G,\mathit{\operatorname{int}}}\times {f}_{u,G}\right)} $$

Where CL_G,int_ is the estimated intrinsic gut wall clearance (whole-organ), f_u,G_ is the fraction unbound in the gut, which was assumed to be 1, and Q_gut_ is the effective blood flow at the gut wall (30) which is defined by:

10 $$ {Q}_{gut}=\frac{Q_{villi}\times {CL}_{perm}}{Q_{villi}+{CL}_{perm}} $$

In which Q_villi_ is the villous blood flow and CL_perm_ is the permeability through the enterocytes of the gut wall for the drug. This permeability factor depends on the effective intestinal permeability per unit surface area (30) (Table I) and the intestinal surface area A described by eq. 12, and can be calculated using eq. 11.

11 $$ {CL}_{perm}={P}_{eff, man}\times A $$

12 $$ \mathrm{With}\ A=2\pi r\left(r+h\right) $$

where r is the intestinal radius in meters and h the intestinal length in meters, which are both calculated using a BSA-based formula (eqs. 13 and 14) (12):

13 $$ \mathrm{r}=\frac{1}{2}\times \left(0.016\times \mathrm{BSA}+0.0159\right) $$

14 $$ \mathrm{h}=2.56\times \mathrm{BSA}+2.95 $$

The total intestinal surface area was cut-off at a maximum value of the adult value of 0.66 m^2^ (30) (figure S2). The total plasma clearance was calculated using eq. 15:

15 $$ {CL}_{plasma}=\frac{Qh\times {CL}_{H,\mathit{\operatorname{int}}}\times {f}_u}{Q_h+\left({f}_u\times {CL}_{H,\mathit{\operatorname{int}}}\right)/\left(B:P\  ratio\right)} $$

Where Q_h_ is the hepatic blood flow, CL_H,int_ is the estimated intrinsic hepatic clearance, f_u_ is the fraction unbound in plasma, and B:P ratio is the blood-to-plasma ratio of midazolam.

### Statistical Model

Inter-individual variability in the estimated intrinsic clearance parameters for midazolam and 1-OH-midazolam was included in the model using the following equation:

16 $$ {CL}_{\mathit{\operatorname{int}},i}={\theta}_{TV}\times {e}^{\upeta_i} $$

In which CL_int,i_ is the individual intrinsic clearance estimate for individual *i*, θ_TV_ is the typical value of the intrinsic clearance in the studied population and η_i_ is a random variable for the *i*th individual form a normal distribution with a mean of zero and variance of ω^2^, yielding a log-normal distribution for the parameter value in the population. Inter-individual variability in the estimated intercompartmental clearance parameters (Q_cp1_ and Q_cp2_) for midazolam was included as well.

Residual unexplained variability was modelled using a combined proportional and additive error model for both midazolam and 1-OH-midazolam. The *j*th observed concentration Y of the *i*th individual was modelled according to:

17 $$ {\mathrm{Y}}_{\mathrm{ij}}={\mathrm{C}}_{\mathrm{pred},\mathrm{ij}}\times \left(1+\upvarepsilon {1}_{\mathrm{ij}}\right)+\upvarepsilon {2}_{\mathrm{ij}} $$

where C_pred,ij_ is the *j*th predicted midazolam concentration of the *i*th individual, ε1_ij_ and ε2_ij_ are random variables from a normal distribution with a mean of zero and variance of σ^2^.

### Covariate Analysis

A covariate analysis for the estimated parameters (whole-organ intrinsic clearance (CL_G,int_, CL_H,int_, CL_H,int,M_) and intercompartmental clearance (Q_cp1_, Q_cp2_)) was performed in which the following covariates were tested for significance: age, body weight, height, body surface area, and sex. For sex, the typical value (θ_TV_) for girls was estimated relative to the value for boys. The remaining continuous covariates were tested using a power (Eq. 18) or linear (Eq.19) function.

18 $$ {P}_i={\theta}_{TV}\times {\left(\frac{COV}{ COV med}\right)}^{\theta_{COV}} $$

19 $$ {P}_i={\theta}_{TV}\times \Big(1+{\theta}_{cov}\times \left( COV-{COV}_{med}\right) $$

where P_i_ is individual parameter estimate for individual *i*, θ_TV_ the typical value of the parameter in the studied population with a median value (COV_med_) of the covariate (COV) and θ_COV_ the estimated exponent or slope for a power or linear function respectively.

### Model Evaluation

Structural models were evaluated by comparison of the objective function values (OFV, i.e. -2 × log-likelihood). A decrease of 3.84 in the OFV between nested models (*p* < 0.05) was considered statistically significant. In addition, goodness-of-fit plots of midazolam and 1-OH-midazolam were assessed, in which observed *versus* individual- and population-predicted concentrations and conditional weighted residuals (CWRES) *versus* time and population predicted concentrations are visualized. Moreover, the condition number, the confidence interval of the parameter estimates, and visual improvement of the individual plots were used to evaluate the models.

For inclusion of covariates, a drop in OFV by at least 6.64 points (*p* < 0.01) was considered statistically significant, while for the backward deletion a more stringent *p* value (*p* < 0.005, ΔOFV>7.88) was used. Moreover, to retain a covariate in the model, the inter-individual variability in the PK parameter should decrease.

Two methods were applied to evaluate the final model internally. A bootstrap analysis (*n* = 250) was performed to evaluate model stability and parameter precision (31). In addition, a normalized prediction distribution error (NPDE) analysis was performed using the NPDE package in R (32). For each observed concentration, 1000 midazolam concentrations were simulated based on the parameter values that were obtained for the original model (Table II). The observed concentrations were compared to the range of 1000 predicted concentrations. The Wilcoxon signed rank test was used to assess the deviation of the observed mean value of the NPDE to the expected value of 0, and the Fisher variance test was used to assess the deviation of the observed variance from the expected value of 1.

### Sensitivity Analysis

A sensitivity analysis was performed to evaluate the assumptions made in the model. For this, it was evaluated what the impact was of a 50% increase or decrease of the parameter values for intestinal length, the fraction unbound and tissue volumes and blood flows on predicted midazolam concentrations and on the estimated model parameters. Additionally, the impact of the assumed fraction absorbed (F_a_) of 1 (29), on the estimated whole-organ intrinsic clearance in the gut wall and liver, and the derived total plasma clearance, was evaluated, as well as a 50% increase or decrease of volume of distribution of the primary metabolite, 1-OH-midazolam.

## Results

In the model as shown in Fig. 1, the intrinsic gut wall clearance was 5.08 L/h (with a relative standard error (RSE) of 16%) and the intrinsic hepatic clearance was 527 L/h (RSE 7%) for a typical individual of 16 kg (Table II). The increase in these whole-organ intrinsic clearance values, reflected by the inclusion of a power function (eq. 18) correlating body weight as covariate to intrinsic clearance with an exponent of 0.807 (RSE 10%) and 0.472 (RSE 16%) for intestinal and hepatic maturation respectively, appeared to be slightly larger in the gut wall than in the liver (Table II). For intercompartmental clearance of midazolam to the first peripheral compartment (with V_p1_ = 55.2 L), body weight was found as a covariate, while no covariate was identified for intercompartmental clearance to the second peripheral compartment (with V_p2_ = 79.1 L). Lastly, a covariate was included in the model correlating weight to intrinsic hepatic clearance of the metabolite with an exponent of 0.651 (RSE 9%). Age, height, body surface area, and sex were not identified as covariates for the estimated parameters. The gut wall metabolism of the metabolite could not be estimated independently due to model instability, and was therefore estimated as a fraction of the gut wall metabolism of midazolam. The bootstrap results confirmed the model stability and the precision of parameter estimates of the model (Table II).

Figure 2a illustrates the relation between body weight and whole-organ intrinsic clearance in the gut wall and the liver. As illustrated in this figure, the intrinsic hepatic clearance was estimated to be around 105 times higher than the intrinsic gut wall clearance for a typical individual of 16 kg, while this factor differed largely between individuals (factor of 5–405, 95%CI). When the gut wall and hepatic intrinsic clearances are expressed per gram of organ, an inverse trend can be observed for hepatic intrinsic CYP3A activity per gram of organ. In Fig. 2b, the intrinsic clearance per gram of organ are plotted *versus* age, which shows a (slight) decrease in hepatic intrinsic CYP3A activity per gram of liver with age, while no change with age is observed for gut wall intrinsic CYP3A activity per gram of small intestine except for a small drop around the age of 4–5 years.

**Fig. 3:**
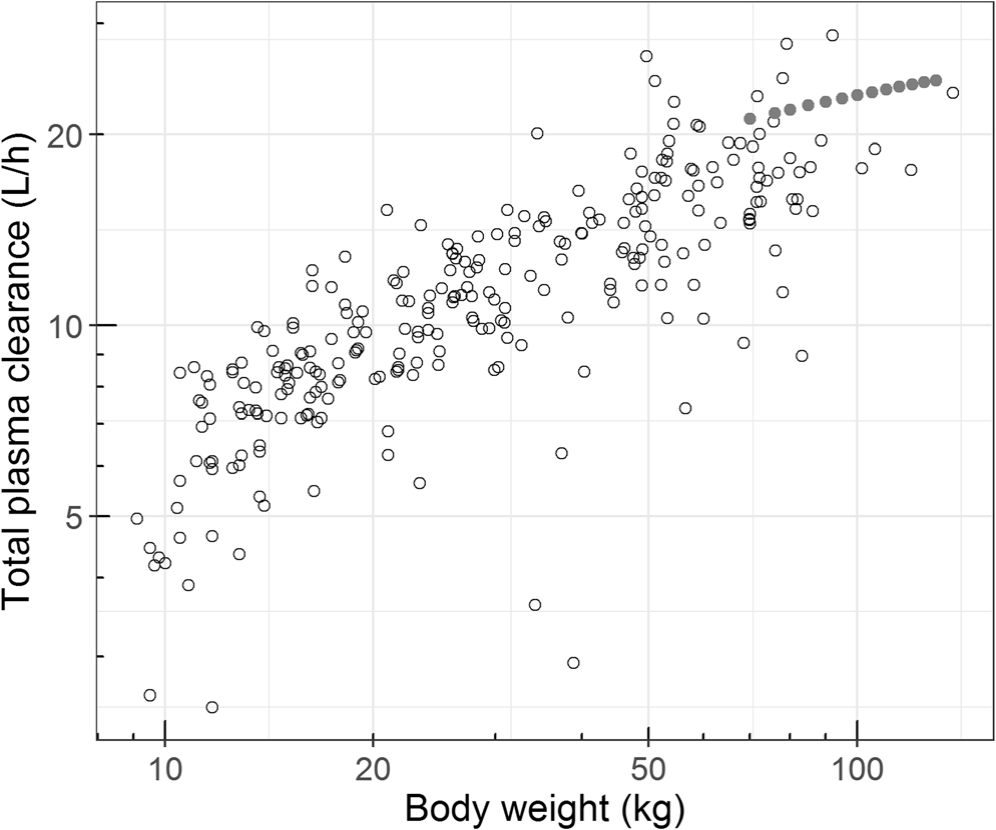
Total hepatic plasma clearance *versus* body weight for children in our study (○) and calculated plasma clearance in adults () based on the reported typical hepatic whole-organ intrinsic clearance of 1640 L/h, a hepatic blood flow increasing with body weight (Q_h_ = 3.75∙WT^0.75^), a fraction unbound of 0.0303 and a blood: plasma ratio of 0.66 (13) using eq. 15.

Using eq. 15, the total plasma clearance was derived and plotted against body weight, showing that total plasma clearance also increases with body weight (Fig. 3). For comparison, in Fig. 2a, b and 3, literature values on whole-organ intrinsic and total plasma clearance in adults have been added (13).

**Fig. 4:**
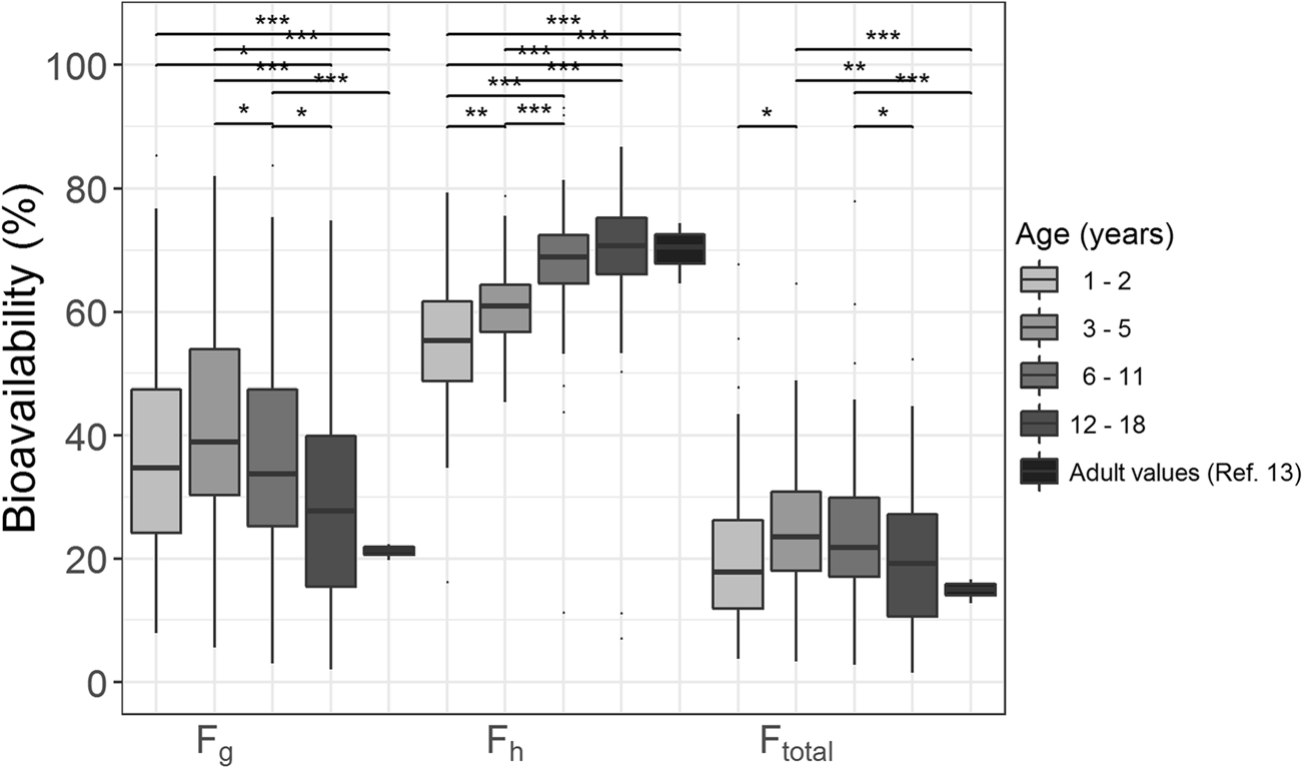
Bioavailability in in the gut wall (F_g_), in the liver (F_h_) and total bioavailability (F_total_) for four different age categories: children of 1–2 years, 3–5 years, 6–11 years, and 12–18 years of age (increasing dark grey) compared to adult values (13). A nonparametric test of group differences was performed using the independent 2-group Wilcoxon-Mann-Whitney Test, with *** indicating a *p*-value <0.001, ** for *p* < 0.01, * for *p* < 0.05 and ‘NS’ for *p* > 0.05. Adult bioavailability values are calculated based on their reported typical whole-organ intrinsic hepatic clearance, hepatic blood flow for their body weight and the fraction unbound (see eqs. 8 and 9) (13).

Using the estimated whole-organ intrinsic clearance values, together with blood flow and fraction unbound, the extraction ratios E_g_ and E_h_ (eqs. 8 and 9) and bioavailability values F_g_ and F_h_ (i.e. 1-E_g_ or E_h_, respectively) were derived. Figure 4 shows the results with gut wall bioavailability first slightly increasing and then decreasing with age, while the hepatic bioavailability showed an overall increase with age. More specifically, the hepatic bioavailability is increasing with body weight from a median of 58% (range 35–79%) to 73% (range 58–86%) from 1 to 2 year old to children 12–18 years of age (*p* < 0.001, Wilcoxon-Mann-Whitney U-test) (Fig. 4). With respect to the gut wall bioavailability, the median of 37% (range 8–85%) in 1–2 year old children increases to 39% (range 6–82%) in children of 3–5 years of age, then to decrease to 34% (range 3–84%) and 29% (range 4–66%) in children of 6–12 and 12–18 years of age, respectively. With total bioavailability being equal to F_g_ times F_h_ (eq. 7), Fig. 4 shows that no or only small changes in the total bioavailability for children up to 12 years of age can be expected. In adolescents, the gut wall, hepatic and total bioavailability proved not significantly different from the values observed in adults which were obtained from literature (*p* > 0.05).

Figure S3 shows the goodness-of-fit plots of both midazolam and its primary metabolite. These plots for midazolam indicated no bias for midazolam in the individual and population concentration predictions *versus* the observed concentrations (figure S3 A,B) and no trend or bias in the conditionally weighted residuals *versus* the predicted concentrations or time after dose (figure S3 C,D). Also the presystemic formation of 1-OH-midazolam was well described by the model, as except for a small under prediction of the peak concentrations (figure S3 E), no bias in the goodness-of-fit plots for the metabolite was observed (figure S3 E-H). The normalized prediction distribution error (NPDE) results showed no bias in the concentration predictions for both midazolam and 1-OH-midazolam (figure S4), indicating no structural model misspecification. The variance of midazolam concentrations however was under-estimated in our model (Fisher variance test, p < 0.001), while the model adequately captures the variability for 1-OH-midazolam concentrations.

The sensitivity analysis (table SII) showed that changing volumes of the gut wall or liver by 50% does not considerably impact the predicted concentrations (<10%) nor our parameter estimates for whole-organ intrinsic clearance for gut wall and liver. With a 50% increase or decrease in hepatic blood flow, the whole-organ intrinsic hepatic clearance inversely changes with −14% and + 49% respectively. A similar trend was found for intestinal blood flow and intestinal length (table SII). A change in fraction unbound in blood resulted in a change in whole-organ intrinsic hepatic clearance by the same factor. If the fraction midazolam that gets absorbed (F_a_) is smaller than 100%, the median total bioavailability does not change considerably. While the hepatic bioavailability is not impacted, the gut wall bioavailability however increases by 4.1 and 18.7% for an F_a_ of 0.90 and 0.80, respectively. A change in the volume of distribution of the primary metabolite leads to considerable changes in all clearance parameters. All results of the sensitivity analysis are summarized in the supplemental material (table SII).

## Discussion

To characterize both intestinal and hepatic CYP3A-mediated metabolism of midazolam in children between 1 and 18 years of age, a physiological population PK model was developed (Fig. 1). The physiological population PK modelling approach we applied has been previously applied in healthy adults (13) and morbidly obese *versus* bariatric surgery patients (14), and this approach has now proven useful in distinguishing between metabolism by intestinal and hepatic CYP3A enzymes in children as well.

Using PK data from one of the few clinical studies in which children varying in age between 1 and 18 years old received midazolam orally (17), we showed that the whole-organ intrinsic clearance of gut wall and liver do not change in parallel (Fig. 2a). For all pediatric ages, the intrinsic hepatic clearance is higher than the intrinsic gut wall clearance, while the gut wall clearance appears to increase slightly faster than hepatic clearance (Fig. 2a). The estimated clearance values for the patients with the highest body weight (>16 years of age) are with 16.1 L/h and 1051 L/h in the same order of magnitude as the reported values of 26.7 L/h and 1640 L/h for intrinsic intestinal and hepatic clearance in adults respectively (13). As we used the whole-organ intrinsic clearance of midazolam as surrogate marker of total hepatic and intestinal CYP3A activity in this study, we found that the total intestinal CYP3A activity is lower than the hepatic CYP3A activity. However, when expressed per gram of organ, no increase in intrinsic gut wall clearance per gram of organ can be observed, while the intrinsic hepatic clearance per gram of liver is highest in the youngest children and decreases with age (Fig. 2b).

The increase in whole-organ intrinsic clearance in the gut wall we report here can be mostly attributed to the organ growth and the increasing total weight of the enterocytes in children (33,34), as per gram of gut wall no trend with increasing age (Fig. 2b) or body weight can be observed in children >2 year of age. The intrinsic intestinal CYP3A4 expression per gram of small intestine in children has been described to be slightly higher in children <2 years of age, compared to 2–5 year old children, and then increases with age (33). This is in agreement with the small drop in gut wall intrinsic CYP3A activity per gram of small intestine we observe around the age of 4–5 years (Fig. 2b), and in combination with the other physiological changes this leads to a higher gut wall bioavailability in children 3–5 year of age compared to children 1–2 years of age, and a decrease in gut wall bioavailability with increasing age in children >3 years of age, which is in agreement with the trend observed in Fig. 4.

We report the whole-liver intrinsic hepatic CYP3A-mediated metabolism to increase with increasing body weight, which can be attributed to an increasing total liver weight (4). In literature, the amount of microsomal protein in the liver is believed to remain constant with age (35), while the CYP3A4 abundance per gram of microsomal protein (8,36) has been reported to increase with age. Our results however show a slight decrease in intrinsic clearance per gram of liver throughout the pediatric age range, suggesting that the absolute abundance or activity of CYP3A4 per gram of liver slightly decreases with age in children 1–18 years of age.

The extraction ratios E_g_ and E_h_ can be derived from the estimated whole-organ intrinsic clearance, the organ blood flows and the fraction unbound (Eqs. 8 and 9), and can be subsequently used to calculate the bioavailability (i.e. F_g_ and F_h_). The fraction escaping gut wall metabolism was found to be smaller (median F_g_ 0.34, range 0.02–0.85) than the fraction escaping hepatic metabolism (median F_h_ 0.66, range 0.35–0.93). These values for hepatic bioavailability are in agreement with previously reported values in adults (13,37). The median hepatic bioavailability increases with body weight due to a smaller increase in whole-organ intrinsic hepatic clearance relative to the hepatic blood flow (Fig. 4), while an inverse, but smaller age-related trend in median intestinal bioavailability was found as the effective blood flow in the gut wall (Q_gut_) increases less with age than the whole-organ intrinsic intestinal clearance.

In adults, the local bioavailability in the gut wall is much lower than in the liver with reported values for F_g_ and F_h_ around 0.2 and 0.7, respectively (13), and therefore in adults the extraction ratio in the gut wall is higher than in the liver, indicating that intestinal CYP3A enzymes play a large role in presystemic metabolism despite the low whole-organ intrinsic clearance. In children, the intrinsic gut wall CYP3A activity is lower than in adults (Fig. 2a), and together with a lower effective blood flow, this leads to a higher gut wall bioavailability compared to adults (Fig. 4). This indicates that the role of gut wall metabolism in presystemic metabolism is smaller in children compared to adults, and also gets smaller with decreasing age. This is compensated by the increased whole-organ intrinsic hepatic CYP3A-mediated intrinsic clearance (Fig. 2a), which together with the hepatic blood flow leads to lower hepatic bioavailability with decreasing age (Fig. 4), resulting in an age-independent total bioavailability F_total_ with a median value of 20.8% (Fig. 4).

The parameters for clearance and total bioavailability are in agreement with literature. For clearance, the plasma clearance can be calculated based on the estimated intrinsic hepatic clearance, the fraction unbound, the hepatic blood flow and the blood-to-plasma ratio (eq. 15). The derived total plasma clearance of 6.0 L/h in the youngest children of 1–2 years of age to 17.5 L/h in the older children ≥16 years of age (Fig. 3), is in agreement with literature values of 0.6 L/h/kg (0.56–0.68 L/h/kg) (28). For patients in the age range between 2 months and 3 years, median clearance values around 9 L/h have been reported in patients after major craniofacial surgery (38) and in patients with severe malaria (39), which is in agreement with our findings. Furthermore, it has been reported that disease severity impacts CYP3A-mediated clearance (40), and as the children in this study are relatively healthy (ASA criterion I or II), clearance is indeed a factor 2–4 higher than the reported midazolam clearance in critically ill children (40–43).

For bioavailability, the median total bioavailability of 20.8% (mean 22.7 ± 12.4%) across the pediatric age range is similar to the reported median value of 13.4% (13) or the reported mean ± SD of 34.3% ± 10% (37) in adults, but the observed variability in total bioavailability in the children in our study was very large, with values ranging from 1.5–77.9% A previous study in pediatric patients ranging in age from 6 months to 16 years, who had ASA physical status I-III, found similar values for bioavailability (28) and also found a large variability with the bioavailability ranging from 9 to 71% (28). This high variability has also been described in other studies (44,45), and may be explained by the high unexplained variability in intestinal CYP3A activity, which may be due to e.g. the circadian rhythm (46), the (genetic) variability in CYP3A5 expression (47), and the different regulation of the gut (11) as a result of exposure to variable food and other environmental effects in different individuals throughout their life (48). This implicates that oral administration will result in highly variable PK profiles and exposure of CYP3A substrates in children, which is relevant in the clinic setting as well as during drug development.

All parameters in the model could be estimated with good precision (RSE < 30%, Table II) and the model stability was evaluated by bootstrap (Table II), which confirmed the precision of the parameter estimates. Furthermore, the goodness-of-fit plots showed that the concentrations of both midazolam and 1-OH-midazolam were well-predicted without bias (figure S3), indicating accurate model predictions for both the parent compound and the metabolite. Figure S4 shows the normalized prediction distribution errors *versus* predicted concentrations and *versus* time after first dose, which demonstrates accurate prediction of the midazolam and 1-OH-midazolam concentrations and variability, with only a small over-prediction of the variability of midazolam.

The model is based on accepted PBPK principles, and well-known equations and parameter values from the literature have been used. For unknown parameter values in the pediatric population, assumptions and scaling methods were applied and a sensitivity analysis was performed for these parameters (Table SII). For hepatic blood flow, the flow was assumed to be a fixed percentage of the cardiac output (25.5% in boys and 28% in girls) (27), which results in blood flow values in agreement with other literature values (49,50). The sensitivity analysis indicated that if the hepatic or the intestinal blood flow would be 50% higher or lower, that peak concentrations would be impacted, but the derived values for extraction ratio and bioavailability were not impacted by this assumption. Moreover, actual blood flows will likely deviate less than 50% from the assumed flows. The assumed tissue volumes (figure S2 C) are in agreement with other literature values (51), and the sensitivity analysis indicated no impact of these assumptions on the results regarding the predicted plasma concentrations and the derived values for extraction ratio and bioavailability. Plasma protein binding to albumin has been accounted for in the model (eq. 2), while no protein binding to other plasma proteins was assumed, and the range of fraction bound was with 96.1–96.4% in agreement with the 97.0% protein binding reported in adults (figure S2 A). Furthermore, our sensitivity analysis indicated that assuming 50% increase or decrease in intestinal length, would lead to +9.3 or − 37.2% change in whole-organ intrinsic gut wall clearance respectively, without affecting the estimated extraction ratio and bioavailability. This is because the intestinal length impacts the permeability factor of the gut wall and thereby the effective blood flow in the gut wall. Since the amount of drug reaching the systemic circulation is derived from the PK data and does not change, an increase in intestinal length is compensated by a decrease in whole-organ intrinsic gut wall clearance so that the extraction ratio and bioavailability remain constant.

The absorption rate constant could not be estimated in our model, as sampling at early time points was limited and therefore we included an absorption rate constant of 4.16 h^−1^, which means that maximum concentrations are reached around 30 min post-dose, which was close to the median observed T_max_ and reported values in literature (28). As in this study only oral midazolam was administered, we could not estimate the volume of distribution for midazolam and its metabolite. We therefore linearly scaled the volumes of the central and peripheral compartments from a 76 kg healthy adult (13) to children. The sensitivity analysis showed that the volume of distribution of the metabolite is impacting all estimated clearance parameters considerably (Table SII), but the assumed distribution volumes are in agreement with previously reported values for volume of distribution in children (28,39). Moreover, since we could not estimate inter-individual variability (IIV) in volume of distribution, all inter-individual variability is in the model attributed to the intrinsic clearance parameters and the residual variability, both of which may therefore be inflated.

Furthermore, we assumed that all midazolam is metabolized by CYP3A into its primary metabolite, 1-OH-midazolam, and the volume and clearance values of the metabolite should therefore be considered as apparent values assuming a 100% formation. As these assumptions on volume of distribution and fraction metabolized are supported by literature (28,39), the PK data of both midazolam and 1-OH-midazolam are well-described (figure S3, S4), and the intrinsic clearance values lead to total plasma clearance and bioavailability in range with previous published work (28,38,39), the intestinal and hepatic intrinsic clearance values are indeed well-estimated by the model and could therefore be used as surrogate marker for gut wall and hepatic CYP3A activity in children. As hepatic CYP3A4 is the most abundance cytochrome P450 enzyme and responsible for metabolism of a wide variety of therapeutics, the observed midazolam clearance as probe for CYP3A activity may have implications for other CYP3A substrates as well. It is also clinically relevant for patients receiving multiple CYP3A substrates or inhibitors/enhancers as different drug-drug interactions in children *versus* adults may be anticipated. However, as the variability in oral bioavailability is very high, a highly variable drug exposure may be anticipated when CYP3A substrates are orally administered.

To conclude, this is the first study in children 1–18 years of age to distinguish between pediatric intestinal and hepatic CYP3A-mediated metabolism using clinical data together with PBPK principles. The results show that the whole-organ intrinsic hepatic clearance appears much higher than the gut wall clearance, but also that the difference between the whole-organ intrinsic clearances in children is smaller compared to adults. As a result, the intrinsic CYP3A-mediated gut wall clearance in children from 1 to 18 years of age contributes less to the overall first-pass metabolism compared to adults. Organ growth is the most important contributing factor to the increase in the whole-organ intrinsic CYP3A clearance in gut wall and liver with age, given the fact that the intestinal CYP3A activity per gram of organ remained relatively constant throughout childhood and the hepatic CYP3A activity per gram of liver even decreased slightly. While intestinal bioavailability decreased with age, the hepatic bioavailability increased with age, resulting in no change in total bioavailability in children with increasing age and body weight. This indicates an age-independent but highly variable first-pass effect by intestinal and hepatic CYP3A enzymes in children from 1 to 18 years of age.

## Electronic supplementary material

ESM 1 (PDF 1.14 MB)
