# Supplementary material for: Characterization of Intestinal and Hepatic CYP3A-Mediated Metabolism of Midazolam in Children Using a Physiological Population Pharmacokinetic Modelling Approach
Source: Pharm Res. 2018 Jul 30;35(9):182. doi: 10.1007/s11095-018-2458-6 (PMC6096899; doi:10.1007/s11095-018-2458-6)
Supplement: Supplementary file 1 — (PDF 1.14 MB) [file 11095_2018_2458_MOESM1_ESM.pdf]

Characterization of intestinal and hepatic CYP3A-mediated metabolism of midazolam in children using a physiological pharmacokinetic modelling approach – Supplemental material

Brussee JM, Yu H, Krekels EHJ, Palić S, Brill MJE, Barrett J, Rostami-Hodjegan A, De Wildt SN, Knibbe CAJ

Supplemental methods

Calculation of height of children was based on age, using formulas from Simcyp® Physiologically-based Pharmacokinetic Modelling and Simulation software [1]. Equations S1 and S2 show the formula for height (HT) in cm based on age in years for boys and girls respectively:

$HT_{male} = 1.76179\times10^{-5}\times age^{7} - 1.19874\times10^{-3}\times age^{6} + 0.0323848\times age^{5} - 0.444112\times age^{4} + 3.2946\times age^{3} - 13.2191\times age^{2} + 33.75\times age + 52.62152$ (eq. S1).

$HT_{female} = -1.51027\times10^{-6}\times age^{8} + 1.21261\times10^{-4}\times age^{7} - 0.0040023\times age^{6} + 0.070179\times age^{5} - 0.708233\times age^{4} + 4.1872\times age^{3} - 14.3393\times age^{2} + 33.84778\times age + 51.535477$ (eq. S2)

The body surface area in m^2 was calculated using equations S3 [2] and S4 [3] for children lower than 15 kg and for children of 15 kg and heavier respectively:

$BSA_{<15kg}\ [m^2] = 0.007184 \times HT[cm]^{0.725} \times WT[kg]^{0.425}$ (eq. S3)

$BSA_{\geq15kg}\ [m^2] = 0.024265 \times HT[cm]^{0.3964} \times WT[kg]^{0.537}$ (eq. S4)

Table SI. Data characteristics

	Densely sampled patients	Sparsely sampled patients	All patients
No. of dosages	31 (1/pt)	233 (1/pt)	264 (1/pt)
No. of samples	327	538	865
Samples/patient	10 (8-11)	2 (1-3)	2 (1-11)
Age (years)	8 (1-17)	7 (1-18)	7 (1-18)
Body weight (kg)	30.2 (9.5-83.2)	26.8 (9.1-137.6)	27.4 (9.1-137.6)
Male/female (%)	15/16 (48/52%)	133/100 (57/43%)	148/116 (56/44%)
Dose (mg)	12.5 (3-15)	10 (3.5-10)	10 (3-15)

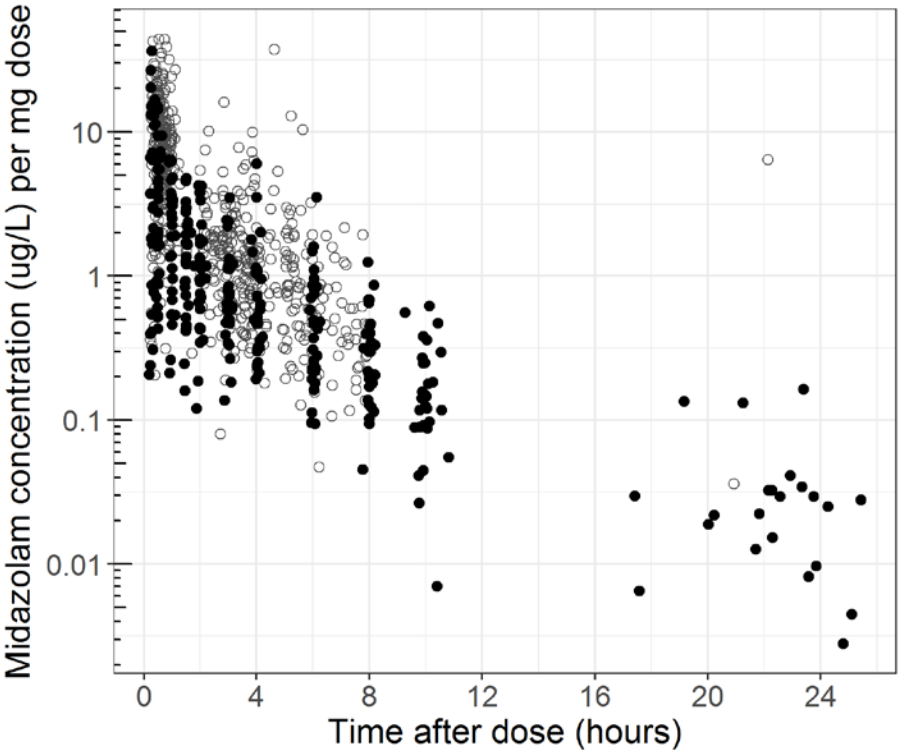

Figure S1. Dose-corrected midazolam concentrations in blood over time for patients who are densely sampled (●) and who are sparsely sampled (○).

Physiological parameters included in the model

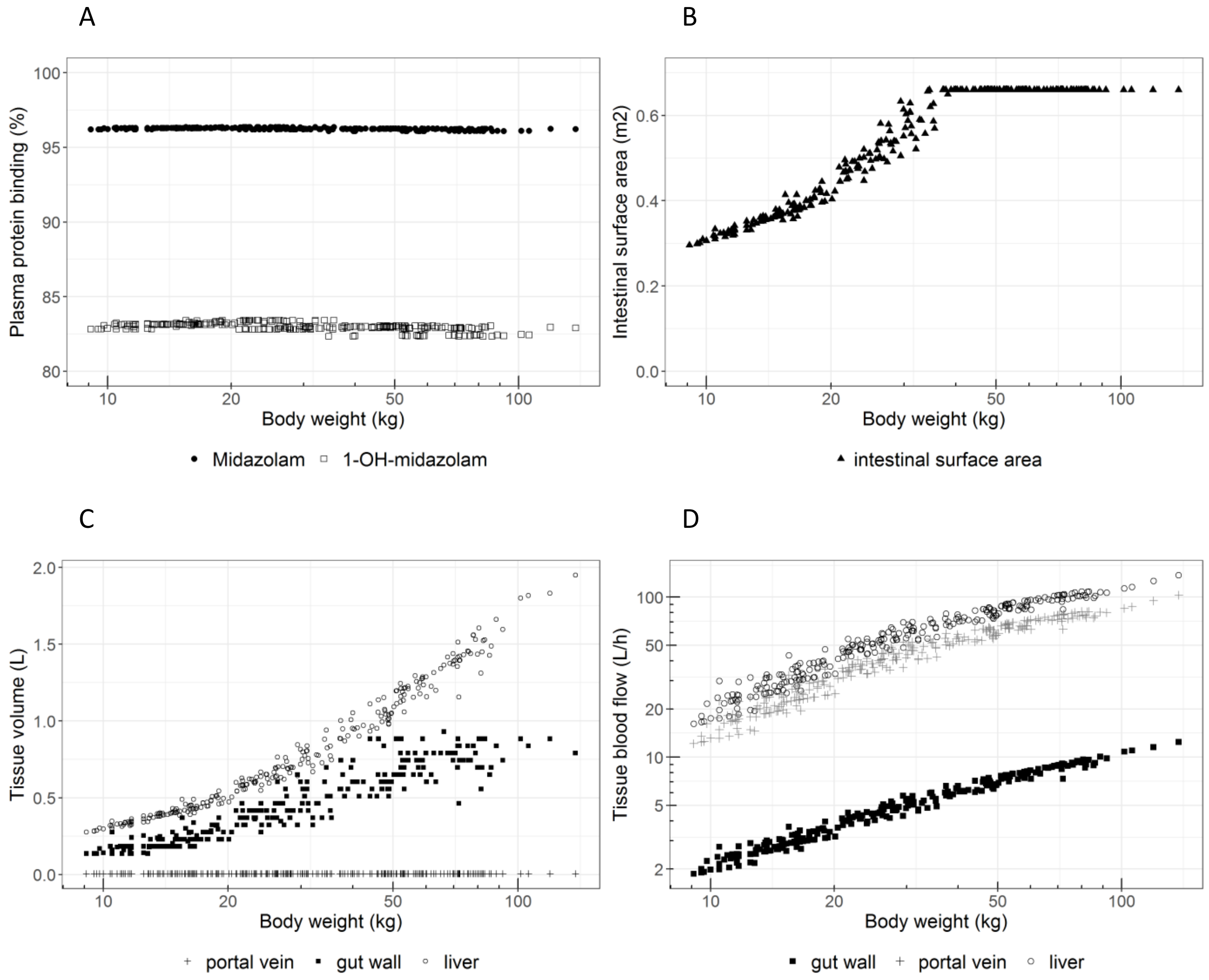

Figure S2. Visualization of relationships between body weight and parameters included in the physiological population PK model for the individuals in the study. For each individual, their actual age, sex, body weight and body surface area are used to calculate the physiological parameters using the formulas from table I, and therefore individuals of the same bodyweight can have different parameter values. **A**) Plasma protein binding for midazolam and 1-OH-midazolam. **B**) Intestinal surface area based on body surface area (eq. 12-14). **C**) Tissue volumes for the gut wall, and liver, which are based on body surface area and age, respectively (eq. 4-5) and the portal vein volume of 5.2 mL. **D**) Tissue blood flows for the gut wall, portal vein and liver, based on cardiac output (eq. 6) and sex. Liver blood flow is the sum of portal vein and hepatic artery flow, which contribute 75% and 25% to the total Q_h, respectively.

Supplemental results

Figure S3 (next page). Goodness-of-fit plots for midazolam (A-D) and its primary metabolite 1-OH-midazolam (E-H). Plots include individual and population predicted concentration versus observed concentration (A,B,E,F) and conditionally weighted residuals (CWRES) versus predicted concentration (C,G) and versus time after dose (D,H).

Figure S3.

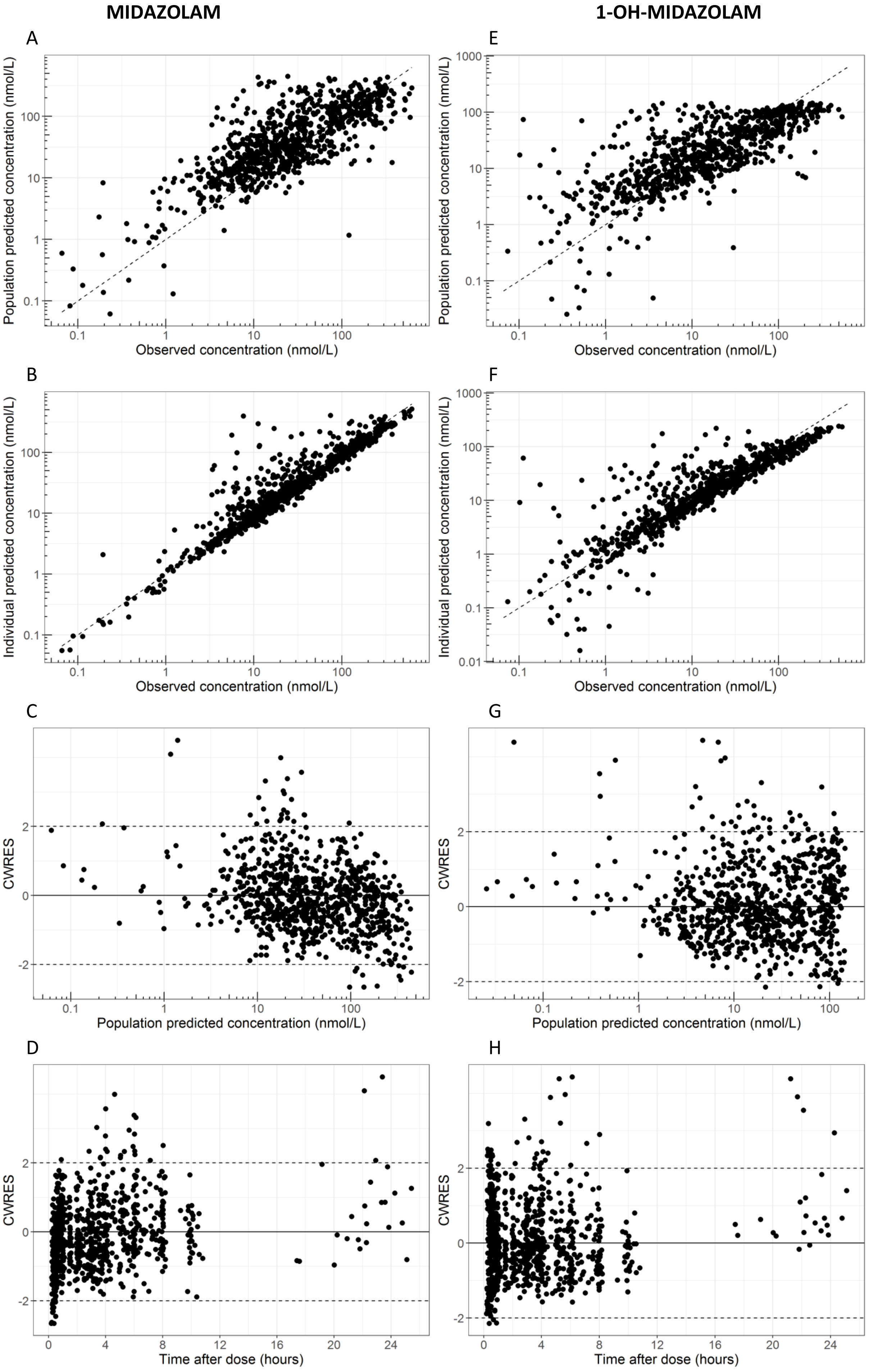
MIDAZOLAM
1-OH-MIDAZOLAM
A
Population predicted concentration (nmol/L)
Observed concentration (nmol/L)
E
Population predicted concentration (nmol/L)
Observed concentration (nmol/L)
B
Individual predicted concentration (nmol/L)
Observed concentration (nmol/L)
F
Individual predicted concentration (nmol/L)
Observed concentration (nmol/L)
C
CWRES
Population predicted concentration (nmol/L)
G
CWRES
Population predicted concentration (nmol/L)
D
CWRES
Time after dose (hours)
H
CWRES
Time after dose (hours)

Figure S4.

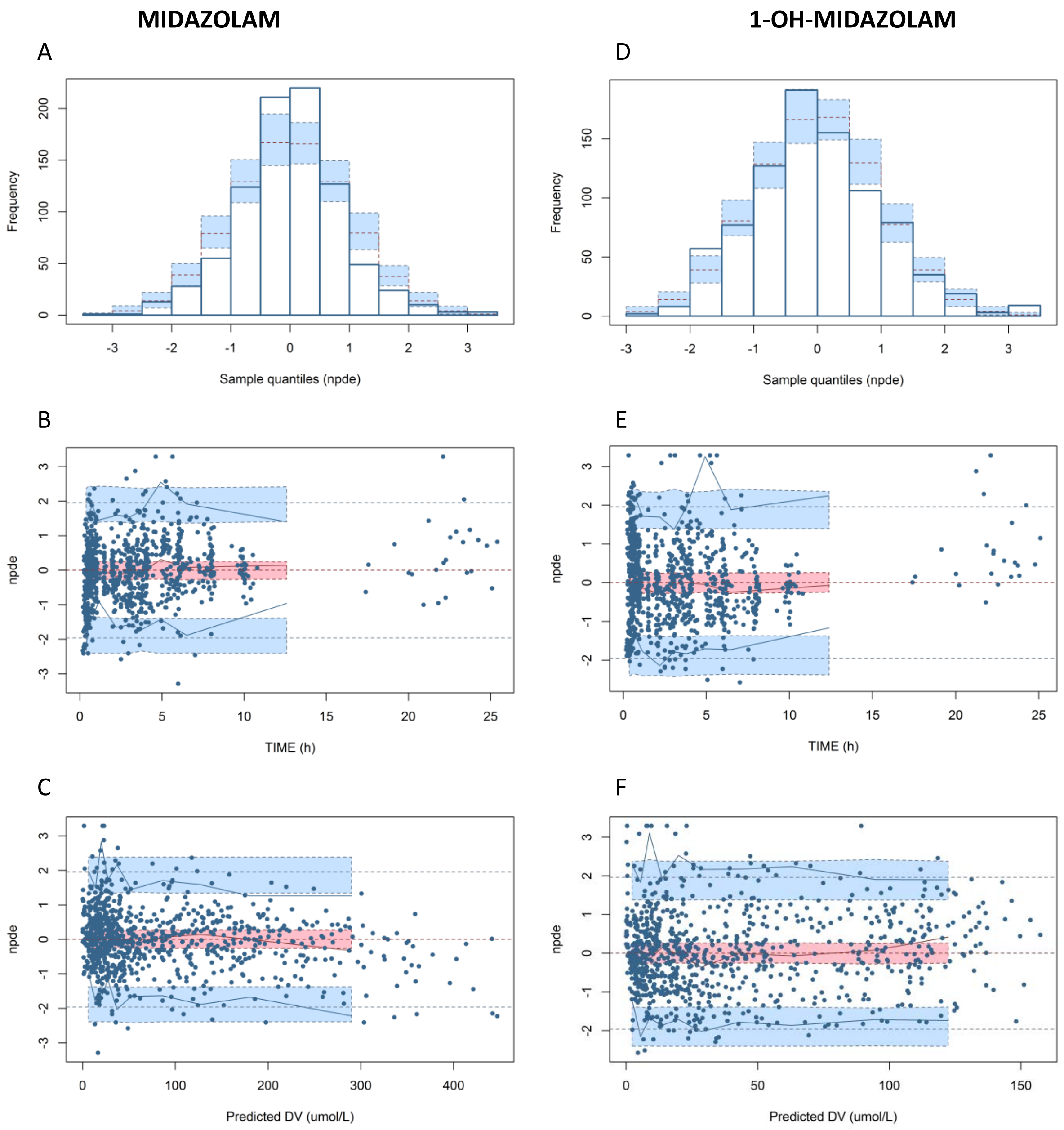

Figure S4. Visualization of the normalized prediction distribution error (NPDE) results for midazolam (**A-C**) and 1-OH-midazolam (**D-F**). First row show the histograms of NPDEs (**A,D**), with the white bars indicating the observed frequency of sample quantiles of the NPDEs, overlaid with the density of the standard normal distribution in blue bars. Second and third row show the NPDE versus time (**B,E**) and versus predicted concentration (**C,F**) respectively, in which the dots represent the NPDE for each observation, the lines indicate the mean (red) and the 95th percentiles (blue) of the NPDEs, and the shaded areas are the simulated 95% confidence intervals of the NPDE median (red) and 95th percentiles (blue).The mean NPDE for midazolam and 1-OH-midazolam were, with 0.003 and -0.004, respectively, not significantly different from 0 (Wilcoxon signed rank test, p>0.1), but the variance for midazolam of 0.76 (p<0.001) was significantly different from 1, while the variance for 1-OH-midazolam of 1.07 (p>0.1) was not significantly different from 1 (Fisher variance test).

Table SII. Results of the sensitivity analysis. The impact on the PK profile of midazolam was evaluated using model simulations, and the impact on the estimated whole-organ intrinsic clearance parameters was assessed based on re-estimation.

		Midazolam PK profile	Intrinsic clearance
**Tissue volumes**	V_h +/-50%	x	$CL_{H,int}$ -5.5%/-17% & $CL_{G,int}$ -16%/+2.0%
	V_{gw} +/-50%	x	$CL_{H,int}$ -1.5%/-7.0% & $CL_{G,int}$ -12%/-11%
**Organ blood flows**	Q_h +/-50%	C_{max} +15%/-40%	$CL_{H,int}$ -14%/+49% & $CL_{G,int}$ +4.7%/-67%
	Q_{vi} +/-50%	C_{max} +15%/-25%	$CL_{H,int}$ -11%/-0.6% & $CL_{G,int}$ +9.5%/-44%
**Intestinal length**	+/-50%	X	$CL_{H,int}$ -14%/-4.8% & $CL_{G,int}$ +9.3%/-37%
**Fraction unbound**	$F_{u,B,prediatric} = F_{u,B,adult}$	X	$CL_{H,int}$ -3.6% & $CL_{G,int}$ +17.2%
**Fraction absorbed**	F_a (0.90)	Full PK profile -10%	$CL_{H,int}$ -15% & $CL_{G,int}$ -3.2%
	F_a (0.80)	full PK profile -20%	CL_{int} -11% & $CL_{G,int}$ -20%
**Volume of distribution 1-OH-midazolam**	V_m +/-50%	X	$CL_{H,int}$ +46%/-27% & $CL_{G,int}$ -41%/+93% & $CL_{H,int,M}$ +43%/-43% & $CL_{G,int,M}$ -55%/+1980%

x: no impact was observed, C_{max}: peak concentrations. CL_{int}: whole-organ intrinsic clearance, H hepatic, G gut wall and M indicating the metabolite 1-OH-midazolam.

References supplemental material

[1] Simcyp® Simulator version 15.1, Certara, Sheffield, United Kingdom, https://www.certara.com/software/physiologically-based-pharmacokinetic-modeling-and-simulation/simcyp-simulator/

[2] Dubois D, Dubois EF. A formula to estimate the approximate surface area if height and weight be known. Archives of Internal Medicine, **1916**, 17: 863-871.

[3] Haycock GB, Schwartz GJ, Wisotsky DH. Geometric method for measuring body surface area: a height-weight formula validated in infants, children, and adults. J Pediatr. **1978** Jul;93(1):62-6.
